# The PUFFinCA scale: development of an instrument for evaluating the primary care functions of family physicians in childhood asthma

**DOI:** 10.1017/S1463423618000609

**Published:** 2018-08-28

**Authors:** Ayşe Nur Usturali Mut, Zeliha Aslı Öcek, Meltem Çiçeklioğlu, Şafak Taner, Esen Demir

**Affiliations:** 1 Public Health Specialist, Public Health Department, Ankara University School of Medicine, Ankara, Turkey; 2 Public Health Specialist, Department of Public Health, Ege University Faculty of Medicine, İzmir, Turkey; 3 Pediatrics and Child Allergy Specialist, Department of Pediatrics, Division of Pulmonology-Allergy, Ege University Faculty of Medicine, İzmir, Turkey

**Keywords:** asthma, family physicians, primary care, reliability, validity

## Abstract

**Aim:**

To develop the Primary care fUnctions oF Family physicians in Childhood Asthma (PUFFinCA) scale for evaluating the cardinal process functions of primary care services (accessibility, comprehensiveness, continuity and coordination) provided by family physicians (FPs) in the management of childhood asthma.

**Background:**

In the literature on the functions of primary care, there is no assessment tool focusing on children with asthma. Primary care assessment scales adapted to various languages are not suitable to adequately address the needs of special patient groups, such as children with asthma.

**Methods:**

In this methodological study, the instrument development process was completed in four stages: establishing the pool of items, evaluating the content validity, applying the scale and statistical analysis. The scale was applied to 320 children who had asthma and received care in the clinic of the Department of Pediatrics, Division of Allergy and Pulmonology at Ege University School of Medicine, Turkey. The Cronbach’s *α* and Spearman–Brown coefficient were calculated to determine the reliability of the scale. Principal component analysis was used to determine the construct validity of the scale.

**Findings:**

The PUFFinCA scale was found to have four-factor structure and 25 items. Cronbach’s *α* coefficient was 0.93. It has been determined that the reliability was excellent and the item-total correlation coefficients were >0.30 each. The factors were titled FP’s ‘functions of accessibility, first contact and continuity’, ‘functions of coordination and comprehensiveness of health services related to asthma management’, ‘provision of preventive care related to asthma’ and ‘provision of services for paid vaccinations’.

## Background

The cardinal functions of primary care can be addressed as three levels, consisting of structure, process and outcome. The structure of a primary care is composed of governance, economic conditions and workforce development. The process of primary care, including dimensions related to the services delivered, consists of four features: accessibility, comprehensiveness, continuity and coordination. The outcome of a primary care is determined by quality, efficiency and equity in health (Kringos *et al*., 2010). The achievement of these core features of primary care indicates that a health system is oriented toward strong primary care services (Kringos *et al*., 2015).

Measurement of the cardinal functions of the process level will determine the extent to which primary care services are accessible regardless of socioeconomic and demographic status, the extent to which the preventive and curative health services offered in the primary care unit cover the needs of the community, whether a long-term or lifelong relationship could be established between the primary care provider and the individuals or the community and the extent to which the information about previous health problems of individuals and about services they received for these problems are integrated by primary care (Starfield, 1998; Kringos *et al*., 2015). Among these functions, although accessibility is fulfilled substantially in many countries, coordination cannot be achieved successfully (Lee *et al*., 2009; Kringos, 2013; Yang *et al*., 2013; Pavlič *et al*., 2015; Akman *et al*., 2017).

Primary care is critical for chronic patients who have to receive continuous service from multiple sources due to the extended period of disease. It has been reported that a robust primary care service in terms of its cardinal functions will have success in determining the risk factors, early diagnosis, treatment and preventing the complications for chronic diseases. Therefore, development of the assessment tools for the cardinal functions of primary care for patients with special needs such as asthmatic children will help to assess the level of these functions and contribute to the control and treatment process of the disease (Starfield, 1998).

Primary care assessment scales adapted to various languages are not suitable to adequately address the needs of special patient groups, such as children with asthma. None of them contain items about preventive care related to asthma such as questioning the environmental, psychological and social factors causing child’s asthma attacks and evaluating home conditions for these factors or giving instructive information notes about what to do when child has an asthma attack (Safran *et al*., 1998; Cassady *et al*., 2000; Seid *et al*., 2001; Shi *et al*., 2001; Mead *et al*., 2008; Lee *et al*., 2009; Rocha *et al*., 2012; Yang *et al*., 2013; Wang *et al*., 2014; Mei *et al*., 2016).

The aim of this study is to develop the Primary care fUnctions oF Family physicians in Childhood Asthma (PUFFinCA) scale for evaluating the cardinal process functions of primary care services (accessibility, comprehensiveness, continuity and coordination) provided by family physicians (FPs) in the management of childhood asthma. The newly developed PUFFinCA scale was used to determine the extent to which the FPs fulfill the process functions when providing healthcare for the children between the ages of 3 and 18 who have asthma and receive care in the clinics of the Department of Pediatrics, Division of Allergy and Pulmonology at Ege University School of Medicine.

## Methods

**Figure 1:**
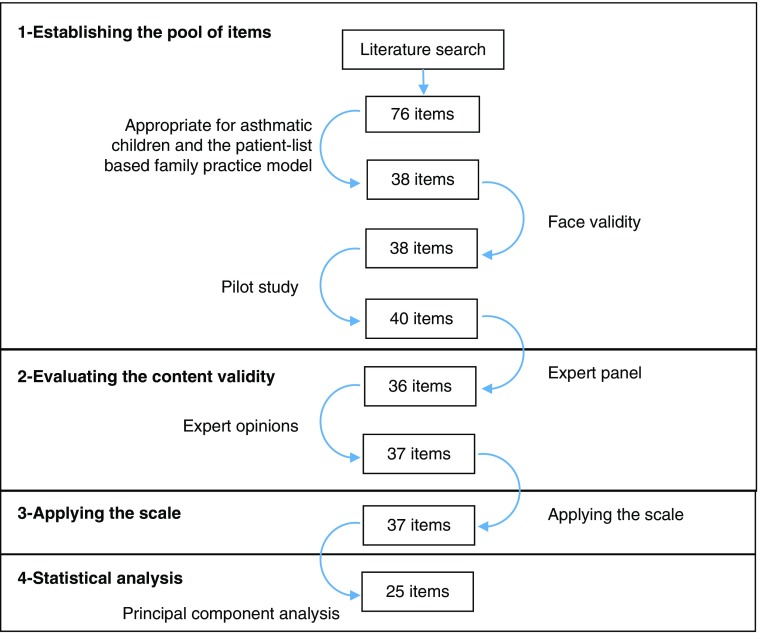
The development process of the Primary Care Functions of Family Physicians in Childhood Asthma scale and the number of items

In this methodological study, the instrument development process was completed in four stages as follows (Figure 1).

### First step: establishing the pool of items

By combining items, statements and questions related the process functions of primary care in the literature, a pool consisted of 76 items was created (Starfield, 1979; Safran *et al*., 1998; Cassady *et al*., 2000; Seid *et al*., 2001; Shi *et al*., 2001; Macinko *et al*., 2007; Mead *et al*., 2008; Lee *et al*., 2009; Diedhiou *et al*., 2010; Schafer *et al*., 2011; Rocha *et al*., 2012; Yang *et al*., 2013; Wang *et al*., 2014; Kringos *et al*., 2015; Wei *et al*., 2015; Mei *et al*., 2016). A group of specialists, including the investigator and three public health specialists, evaluated the appropriateness of these items for asthmatic children and the patient-list-based family practice model. All of the existing questionnaires and scales were on general health needs and not developed specifically for childhood asthma. In particular, there were 11 general items not related to asthmatic children but related to comprehensiveness function such as cancer prevention and screening, family planning and drug/alcohol counseling. In addition, there were 27 duplicate items. After these items were removed, 38 new Turkish sentences were generated based on the remaining 38 items and evaluated for face validity. After a pilot study with five mothers who have children with asthma, two more items were added.

### Second step: evaluating the content validity

- Expert panel: The investigator, two public health specialists, a specialist in pediatric pulmonary diseases and allergy, a FP and a mother of a child with asthma participated in the expert panel to evaluate the content validity. It has been decided in the panel that the expression of 11 items were revised, six items were removed, two new items were added and the items were answered with one of the following options: always, usually, sometimes, rarely and never.
- Expert opinions: The 36-item scale formed in the expert panel was evaluated in terms of content validity by a group of 13 experts, including five FPs, five public health specialists, two specialists in pediatrics and a specialist in pulmonary diseases. This evaluation graded each item as: ‘1=not suitable’, ‘2=the item needs to be changed to be suitable’, ‘3=fairly suitable but small changes may be required’ and ‘4=very suitable’ followed by suggestions if there is any. The options ‘3=fairly suitable but small changes may be required’ and ‘4=very suitable’ were considered suitable and the content validity ratios (CVR) were calculated. Accordingly, it has been found that the CVR values of the two items were lower than 0.54, the minimum CVR value recommended by Lawshe for 13 evaluators (Lawshe, 1975). Since these two items, which express the out-of-hours accessibility and the economic accessibility, were important components of the related scales in the literature, they were not removed but re-worded. An item has been added in light of the recommendations of the experts.

### Third step: applying the scale

The scale was applied to the person accompanying the child who had asthma and received care in the clinic of the Department of Pediatrics, Division of Allergy and Pulmonology at Ege University School of Medicine, Turkey. In order to represent the number of patients, the patient files in the clinic archive were examined based on the criteria of receiving asthma diagnosis and visiting the clinic in the last five years, and the population size of the study was found to be 650 children. The sample size was calculated as 312 in Epi Info Software using a prevalence of 50%, a confidence interval of 95% and a margin of error of 4% for a population size of 650. This number also meets the principle that requires including five people for each item in the scale and the total number of people to be at least 100 (Streiner, 1994). Since all participants were interviewed by the same person, there was no need to examine inter-rater reliability. During the data collection process, 334 children were admitted to the clinic and 320 children were included in the study group. Fourteen children were not included in the study group, because five children did not accept to participate in the study, seven children were not eligible according to the inclusion criteria and three people accompanying the children did not want to answer some of the items in the scale. Inclusion criteria were as follows: applying to the clinic between 02/16/2016 and 07/15/2016, having an asthma diagnosis by the physician, being between the ages of 3 and 18 years at the time of application, the presence of an adult beside the child, who knows the child’s health problems and asthma-related services received and not having a sibling included in the study before. Childhood asthma is defined as physician diagnosed asthma in children who were older than 3 years and younger than 18 years in this study.

### Forth step: statistical analysis of the scale findings in terms of reliability and validity

The data set consisting of 320 children was analyzed. There were no missing data. The Cronbach’s *α* coefficient was calculated to determine the reliability of the scale; the split half (calculation of Spearman–Brown coefficient) and item-total correlation method were used; and the changes in all means or the reliability coefficients if an item deleted were evaluated. Exploratory factor analysis (principal component analysis) was used to determine the construct validity of the scale. The varimax rotation method, an orthogonal rotation method, was applied; Bartlett’s sphericity test *P*-value, Kaiser–Meyer–Olkin (KMO) value and the measure of sampling adequacy (MSA) were calculated. An eigenvalue >1.0 was used as the criterion for determining the number of factors. Factor loadings were required to be >0.4 and the items with factor loading difference of 0.1 or below under multiple factors were removed from analysis (Field, 2005).

In producing the total and sub-dimension scores of the newly developed scale, the scoring was: ‘always’=5 points, ‘usually’=4, ‘sometimes’=3, ‘rarely’=2 and ‘never/not suitable’=1. The scores for the answers given to the items belonging to the sub-dimensions were summed and the scores of all the children constituting the study group were obtained by dividing the sum by the number of items.

## Results

The KMO value was excellent (0.92) and Bartlett’s sphericity test *P-*value was significant (<0.001). The MSA values of all items were >0.50 and suitable for factor analysis. Principle component analysis indicated that the factor loading differences of 12 items under multiple factors were 0.10 or below. As a result of factor analysis done by sequentially removing these items, the PUFFinCA scale was found to have four-factors structure and 25 items. The eigenvalues of the factors, the percentages of total variance explained by the factor, the cumulative percentage of the total variance and the lowest and highest values for the factor loadings of the items in the factors are presented in Table 1.

**Table 1:** Factor structure resulting from factor analysis of the scale

Factor	Eigenvalue	% of total variance	Cumulative % of total variance	Factor loading
Factor 1	10.8	25.8	25.8	0.937–0.652
Factor 2	4.4	24.8	50.7	0.846–0.578
Factor 3	1.1	12.4	63.2	0.753–0.583
Factor 4	1.0	6.5	69.7	0.878–0.610

Cronbach’s *α* coefficient of the PUFFinCA scale was 0.93 and the Spearman–Brown coefficient was 0.93. It has been found that the reliability was excellent and the item-total correlation coefficients were >0.30 each. The factors were titled ‘FP’s functions of accessibility, first contact and continuity’, ‘FP’s functions of coordination and comprehensiveness of health services related to asthma management’, ‘FP’s provision of preventive care related to asthma’ and ‘FP’s provision of services for paid vaccinations’ (Table 2). The average score calculated for the responses given by the study group to the PUFFinCA scale items was 2.33±0.70. The sub-dimension of the accessibility, first contact and continuity functions has the highest average scores, whereas the sub-dimension of providing preventive care for asthma has the lowest average score (Table 3).

**Table 2:** Items related to the factors of the Primary Care Functions of Family Physicians in Childhood Asthma scale and analysis results for the items

Items	Mean score	SD	Factor loading	Item-total correlation coefficient	Spearman–Brown coefficient	Cronbach’s *α* coefficient
Factor 1: Functions of accessibility, first contact and continuity					0.916	0.945
1: I go to the FP first when my child has health problems other than asthma (sore throat, fever, vomiting, etc.)	3.21	1.36	0.652	0.691		
2: I can easily access FP on foot or by car	4.05	1.21	0.831	0.813		
3: I can see the FP or talk over the phone on the same day when I need advice about my child’s health during office hours	3.38	1.42	0.727	0.686		
4: The payments we make for visits to the FP for my child and the prescribed drugs do not create a burden on our budget	3.60	1.35	0.686	0.561		
5: The FP allocates sufficient time for me when I go to his/her office for my child	3.81	1.38	0.856	0.860		
6: I can easily ask the FP about anything that comes to mind when I visit his/her office for my child’s problem	4.03	1.37	0.929	0.917		
7: If there is anything I do not understand in the FP’s explanations I can ask him/her to repeat	4.06	1.37	0.937	0.899		
8: I think the FP understands me when I say or ask something about my child	3.99	1.38	0.918	0.887		
9: When necessary, the FP refers to other healthcare facilities and physicians related to my child’s asthma	3.75	1.46	0.765	0.775		
Factor 2: Functions of coordination and comprehensiveness of health services related to asthma management					0.905	0.917
1: The FP informs the specialist we visit for asthma about my child’s health, the examinations previously done, etc.	1.33	0.72	0.613	0.605		
2: After my child has received care related to asthma from specialist, the FP talks to me about what has been done in that visit	2.32	1.24	0.597	0.702		
3: The FP wants to see the results of the examinations conducted at other health institutions for asthma and all the documents related to my child’s health	1.66	1.05	0.846	0.854		
4: The FP records the results of these examinations and the health-related documents	1.46	0.87	0.832	0.839		
5: The FP explains to me the results of these examinations in a way that I can understand	1.70	1.21	0.844	0.817		
6: The FP demonstrates how my child will use asthma devices and medications	1.68	1.00	0.578	0.668		
7: The FP calls my child to check for asthma at regular intervals, even if he/she does not have asthma attack	1.16	0.49	0.716	0.617		
8: If/when my child has asthma attack, I receive emergency care service for this attack from the FP	1.44	0.80	0.687	0.671		
9: The FP gives us/caregiver/teacher information notes about what to do when my child has an asthma attack	1.42	0.75	0.735	0.768		
Factor 3: Provision of preventive care related to asthma					0.690	0.867
1: The FP gives information about the factors causing my child’s asthma attacks	1.86	1.14	0.725	0.867		
2: The FP questions the environmental (allergens, cigarette smoke, air pollution, infections, drugs, foods, food additives and exercise), psychological (stress and emotional changes) and social factors that cause my child’s asthma attacks	1.71	1.10	0.753	0.915		
3: The FP questions the domestic factors causing my child’s asthma attacks	1.67	1.08	0.741	0.905		
4: The FP or nurse/midwife working with him/her visits our home and evaluates the home conditions for factors that cause asthma attacks	1.10	0.43	0.591	0.445		
5: The FP measures height and weight of my child to follow his/her growth and development	1.38	0.91	0.583	0.421		
Factor 4: Provision of services for paid vaccinations					0.707	0.707
1: I get my child’s influenza/pneumonia vaccines done at the FP center	1.52	1.10	0.878	0.546		
2: The FP gives information and suggestions about special vaccines that my child should get such as influenza and pneumonia	1.89	1.10	0.610	0.546		

FP=family physician.

**Table 3:** Mean, SD, minimum and maximum values for the total scale score and the factor scores

Factors of the scale	Mean	SD	Minimum	Maximum
Functions of accessibility, first contact and continuity	3.83	1.16	1.00	5.00
Functions of coordination and comprehensiveness of health services related to asthma management	1.57	0.72	1.00	4.67
Provision of preventive care related to asthma	1.54	0.78	1.00	4.40
Provision of services for paid vaccinations	1.70	0.97	1.00	5.00
Total score	2.33	0.70	1.00	4.54

## Discussion

The results of the study showed that the newly developed PUFFinCA scale was successful in terms of validity and had four sub-dimensions: 1=FP’s functions of accessibility, first contact and continuity; 2=coordination and comprehensiveness of healthcare services; 3=preventive care services and 4=the services related to paid vaccines. The total variance explained by the instrument consisting of 25 items was found to be 69.70%, and the reliability was excellent.

Considering its dramatically increasing prevalence and features requiring a holistic management process, childhood asthma is a very representative disease to assess the existing level of process functions of primary care. Furthermore, determining of problematic points of primary care system by means of a scale developed specifically for childhood asthma provides more meaningful information for policymakers (Connor and Mullan, 1983; Starfield, 1998; Wagner *et al*., 2001). The PUFFinCA scale is the first scale developed specifically for asthmatic children and evaluating the basic functions of FPs. It was therefore compared with other scales that include the cardinal process functions of the primary care in terms of psychometric properties.

When various primary care evaluation scales were examined, in a study of primary care assessment scale, Shi *et al.* found the variance explained by seven factors to be 88.10%. The first contact function was evaluated by seven items in this study, continuity by 20, coordination by 8 and comprehensiveness by 34 items (Shi *et al*., 2001). The instrument that was created in the United States to assess the functions of primary care for children consisted of five sub-dimensions, and had an explained variance of 48% and Cronbach’s *α* coefficient of 0.40–0.81. The sub-dimensions of the scale were continuity, first contact – accessibility, comprehensiveness – services available, comprehensiveness – services provided and coordination (Cassady *et al*., 2000). In another study evaluating the primary care in terms of the services offered to children, the total variance explained by the sub-dimensions of continuity, accessibility, information, communication, comprehensiveness and coordination was found to be 77% (Seid *et al*., 2001). In a study evaluating the primary care for adults in South Korea, the scale with five sub-dimensions was found valid and reliable. The sub-dimensions of this scale were determined as individual-oriented care, the coordination function, comprehensiveness, family/community orientation and first contact (Lee *et al*., 2009). In the primary care evaluation study by Safran *et al*. (1998), the Cronbach’s *α* coefficient of the scale with 11 sub-dimensions was found to be 0.81–0.95. Similarly, other studies involving the development and adaptation of various primary care evaluation scales had number of factor ranging from 2 to 9 and explained variance ranging from 56.40 to 73% (Mead *et al*., 2008; Rocha *et al*., 2012; Yang *et al*., 2013; Mei *et al*., 2016). The PUFFinCA scale is said to be similar to other studies in terms of the number of factors, the total variance explained and the reliability coefficient. On the other hand, inclusion of items related to the preventive care services for children with asthma collected in two sub-dimensions was a distinctive feature of this scale. In this respect, the PUFFinCA scale may guide the evaluation of the needs of asthmatic children.

The number of items in scales is of critical importance for ease of application. The scales developed in previous studies and the number of items contained are as follows: Shi *et al*. had 92 items; Safran had 51, Cassady had 33, Seid had 23 and Lee had 21 items; comparable scales had number of items varying between 10 and 34 (Safran *et al*., 1998; Cassady *et al*., 2000; Seid *et al*., 2001; Shi *et al*., 2001; Mead *et al*., 2008; Lee *et al*., 2009; Rocha *et al*., 2012; Yang *et al*., 2013; Mei *et al*., 2016). In our study, we found that 12 of the 37 items had similar factor loadings under the different factors. It can be said that the remaining 25 items compensated for the discarded items to a large extent and that the PUFFinCA scale was fairly suitable in terms of the number of items.

When the sub-dimension scores were examined, accessibility, first contact and continuity functions (3.83) were found to have higher scores than the other three sub-dimensions, which had just acceptable scores. In the two scales developed by Lee *et al*. (2009) and Yang *et al*. (2013), accessibility sub-dimension had higher scores than coordination and comprehensiveness. In a study involving 31 European countries, accessibility, continuity and comprehensiveness had good higher scores, whereas coordination had relatively lower scores in the majority of countries (Kringos, 2013). In a study carried out in Turkey, FPs were found unable to fulfill the coordination function (Akman *et al*., 2017). Given that the situation was similarly challenging in many of the countries in terms of coordination, this may be an expected result (Kringos, 2013; Pavlič *et al*., 2015). With respect to the third and fourth sub-dimensions, primary care physicians seem to be unable to adequately provide the preventive care services, their basic roles in managing asthma (Yawn, 2011). Similarly, a study evaluating 28 countries in Europe has also reported a decline in the delivery of preventive care services (Schäfer *et al*., 2016).

It is a longstanding discussion whether a primary care system should be problem-oriented or goal-oriented in terms of chronic disease management (De Maeseneer, 2012). If problem-oriented model is implemented alone, the functions of continuity and comprehensiveness cannot be achieved (Starfield, 1998; De Maeseneer, 2012). Although being a disease-specific scale, PUFFinCA was not developed according to a problem-oriented approach. In order to strengthen its goal-orientedness, we particularly included general items in the first sub-dimension of the scale. Furthermore, asthma has very appropriate characteristics for primary care to be evaluated with a goal-oriented approach; it is common in childhood; it has to be managed with a holistic approach; and it is a chronic disease that can be easily followed in primary care settings. Consequently, the evaluation of primary care in terms of the management process of childhood asthma reflects the management process of other chronic diseases in primary care.

This study has some potential limitations. In the third step of instrument development, the scale was applied to the person accompanying the child receiving care in a university hospital. Therefore, it can be said that developing the tool in tertiary care can cause bias. There is no obstacle to visiting tertiary healthcare institutions in Turkey, because there is currently no referral chain and all Turkish children have health insurance coverage. Considering that all children with asthma can visit any healthcare institutions, it can be said that the children visiting a university hospital do not have very different characteristics in terms of functions of primary care comparing with asthmatic children visiting other hospitals. On the other hand, it can be thought that the study population can have different characteristics compared with the asthmatic children visiting only their FPs. If we had carried out this study in a primary care center, this would have also caused bias differently and would not have represented asthmatic children who never visited primary care. Therefore, it would be appropriate for the third step of instrument development to be repeated in a way that covers the children visiting either primary, secondary or tertiary care settings, that is, to be carried out on a community basis.

The study was based on self-reporting. There can be recall bias because asthmatic children receive continuous service from multiple sources due to the extended period of disease. Parents are often more cautious about childhood illnesses and the items of PUFFinCA scale are not very hard to remember. In addition, there can be response bias. Because this study was not carried out in primary care centers, it was very unlikely that the items about FPs were answered incorrectly. The functions of accessibility, continuity, coordination and comprehensiveness were not collected in separate sub-dimensions. This is an expected result because these functions are highly related to each other. Test–retest reliability was not assessed for the PUFFinCA scale. However, other analyses indicating the reliability of the scale were excellent. Even though PUFFinCA scale was developed in Turkey, it can be used for asthmatic children in all countries after local translation and cultural adaptation.

## Conclusion

Despite these limitations, the PUFFinCA scale, which has satisfactory methodological results, may potentially meet the need for an assessment tool to evaluate the cardinal functions of the FPs particular to asthma. The scale does not contain only outcomes particular to a disease, and it also demonstrates the status of the primary care system regarding chronic disease management by means of a typical disease. In strengthening primary care systems in terms of chronic disease management, valid and reliable instruments will be instructive guide. The outcomes of instruments to be developed will lead to the adoption of a multidisciplinary team approach, definition of different components of primary care teams and development of coordination mechanisms in primary care.
